# Cathepsin K‐Positive Cell Lineage Promotes In Situ Dentin Formation Controlled by Nociceptive Sonic Hedgehog

**DOI:** 10.1002/advs.202310048

**Published:** 2024-10-30

**Authors:** Ruoshi Xu, Xiaohan Zhang, Weimin Lin, Yushun Wang, Danting Zhang, Shuang Jiang, Linfeng Liu, Jiaying Wang, Xutao Luo, Xiao Zhang, Junjun Jing, Quan Yuan, Chenchen Zhou

**Affiliations:** ^1^ State Key Laboratory of Oral Diseases & National Center for Stomatology & National Clinical Research Center for Oral Diseases West China Hospital of Stomatology Sichuan University Chengdu 610041 China

**Keywords:** endodontics, genetic mouse models, homeostasis, regeneration, translational medicine

## Abstract

Oral diseases affect nearly half of the global population throughout their lifetime causing pain, as estimated by the World Health Organization. Preservation of vital pulp is the therapeutic core as well as a challenge to protect natural teeth. Current bottleneck lies in that the regenerative capacity of injured pulp is undetermined. In this study, we identified a lifelong lineage that is labelled by cathepsin K (Ctsk) contributing to the physiological, reactionary and reparative odontogenesis of mouse molars. Ctsk^+^ cell‐mediated dentin formation is regulated by nociceptive nerve‐derived Sonic Hedgehog (Shh), especially rapidly responsive to acute injury. Notably, exogenous Shh protein to the injury pulp can preserve Ctsk^+^ cell capacity of odontogenesis for the nearby crown pulp and even remote root apex growth, alleviating conventionally developmental arrest in youth pulpitis. Exposed to chronical attrition, aged pulp Ctsk^+^ cells still hold the capacity to respond to acute stimuli and promote reparative odontogenesis, also enhanced by exogenous Shh capping. Therefore, Ctsk^+^ cells may be one of the lineages for accelerating precision medicine for efficient pulp treatment across ages. Shh application can be a candidate for vital pulp preservation and pulp injury repair by promoting regenerative odontogenesis to a certain extent from young adults to older individuals.

## Graphical abstract

Mouse molar pulp Ctsk^+^ cells contribute to neonatal odontogenesis and reactionary dentin formation even if aging. Nociceptive nerve, more than conducting pain, secrets Shh to promote such process. Exogenous Shh reactivates Ctsk^+^ cells and expands the limit of pulp self‐repairing capability to enhance reparative dentin, even apex development, hopefully shedding a light on pulp preservation therapeutics covering all age groups.

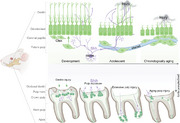

## 1 Introduction

The World Health Organization Global Oral Health Status Report (2022) estimated that oral diseases affect close to 3.5 billion people worldwide across all age, which poses a major health burden for many countries and impacts people throughout their lifespan, causing pain, disfigurement and even death.^[^ ^1^, ^2^ ^]^ Dental caries is the most widespread noncommunicable disease, ranking first for decay of permanent teeth (2.3 billion people) and 12th for deciduous teeth (560 million children).^[^ ^3^ ^]^ According to the United Nations, one in six people globally will be over the age of 65 by 2050,^[^ ^4^ ^]^ foreseeing the huge needs of oral health from the older adults. Vital pulp preservation is of top priority for protecting natural teeth, the indications of which are extremely controlled due to limited success rate clinically. Current therapeutics are limited in their narrow treatment sites, varying levels of pulp inflammation, and unpredictable pulp reactivity among patients of different ages, resulting in restricted indications and limited success rates.^[^ ^5^ ^]^ An economical and highly effective clinical treatment is required for vital pulp preservation that is appropriate for patients from young to older adults.

Dentin injury repair is controlled by a sophisticated regulatory network of odontogenesis. Vital pulp is an essential prerequisite for active odontogenesis, which is innate during development.^[^ ^6^, ^7^ ^]^ In mouse models, dynamic odontogenesis promotes rapid crown growth of the first molar postnatally, particularly from P6.5 to P8.5, and subsequently promotes root dentin initiation, elongation, and thickness until ≈1 month, followed by dentin homeostasis.^[^ ^7^, ^8^ ^]^ Odontogenesis is attributed to functional odontoblasts and their progenitors, which provide possibilities for dentin regeneration. Current literatures reveal that the implantation of human deciduous pulp stem cells into the injured teeth of patients triggers dentin formation and fuels root growth.^[^ ^9^ ^]^ Multipotent dental pulp regenerative stem cells (MDPSCs) express CD24a, a developmental marker detected in the primary dental papilla during odontogenesis.^[^ ^10^ ^]^ ALX homeobox 3‐Wnt family member 3A (Alx3‐Wnt3a), a pivotal factor in dentin development, promotes the innate regenerative capacity of adult tissues to regenerate dentin to the native structure, providing evidence that development and injury repair may share some common regulatory mechanisms. Studying innate developmental mechanisms, which might provide insights to reveal self‐healing of dental pulp, hopefully broadening horizons in this field.

Among dental pulp heterogeneity, which lineage have the significant effect to dentin formation during development and injury repair? The cysteine proteinase Ctsk is secreted by osteoclasts, induces potent bone resorption activity,^[^ ^11^ ^]^ and has been identified as an osteogenic stem or progenitor cell marker.^[^ ^12^ ^]^ In a bicortical fracture mouse model, Ctsk^+[^ ^13^ ^]^ and Gli1^+[^ ^14^ ^]^ periosteal stem cells primarily contributed to repair. Liver kinase b1 (Lkb1) deletion in Ctsk‐expressing periosteal cells promotes osteogenic tumors,^[^ ^15^ ^]^ further confirming that Ctsk^+^ cells function as mesenchymal progenitors. A conditional suppressor of fused (Sufu) knockout in Ctsk‐Cre‐expressing cells was shown to induce spontaneous and progressive heterotopic ossification of the tendon, providing further evidence for Ctsk‐labelled progenitor cells.^[^ ^16^ ^]^ A Ctsk^+^ periosteal subset has been identified in the human jawbone, and a unique Ctsk^+^ lymphocyte antigen 6 complex, the locus A (Ly6a^+^) subset of cells, was proposed to contribute to mouse jawbone repair defects.^[^ ^17^ ^]^ Further, the descendants of a keratin 14^+^ (Krt14) Ctsk^+^ cell subset exhibited dual epithelial and mesenchymal characteristics, contributing to osteogenesis in a maxillary sinus floor lifting mouse model.^[^ ^18^ ^]^ Calvarial Ctsk^+^ lineage interacts among multi‐stem cells in calvaria ossification.^[^ ^19^ ^]^ We previously found calvarial Ctsk^+^ lineage was regulated by Hedgehog signaling and external Hh activator to suture locally partially rescued the weakened osteogenesis,^[^ ^19^ ^]^ leading us to hypothesize that Ctsk‐labelled population may inhabit in dental pulp as a starting point to explore cell‐based pulp preservation strategies.

Here we present a cell lineage labelled with Ctsk that contributes to physiological and injury‐repair odontogenesis during the development and aging of mouse molars, regulated by nociceptive‐nerve derived Shh. Shh‐capping of exposed pulp could preserve the dentin formation capacity of the crown to some extent, even during root development, by activating Ctsk^+^ cells in the crown and apex. Aging reduces the amount of Ctsk^+^ cells but preserves their reactivity to Shh and their dentin formation capacity when acutely injured. Shh‐regulated Ctsk^+^ cells might be the hub to connect the development and injury repair of odontogenesis, suggesting that dental pulp across ages possesses the chance of preserve vitality.

## 2 Results

### 2.1 The Ctsk^+^ Population is Present in the Molar Pulp from Neonatal to Aged Mice

**Figure 1:**
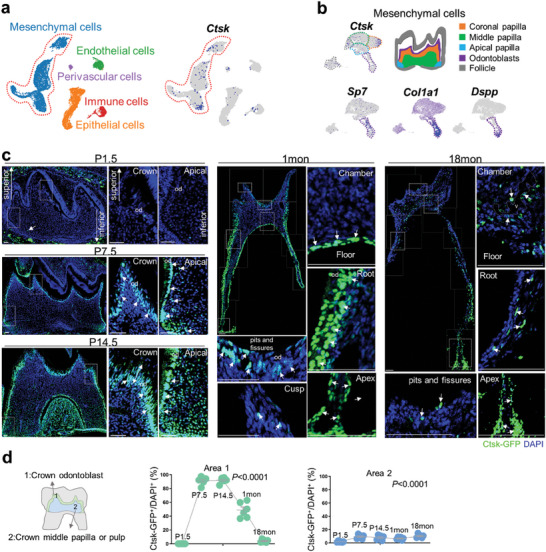
Ctsk expression in mouse molar pulp. a) A UMAP plot of cell types in the mouse molar at P7.5. b) The cluster of mesenchyme from a was further analyzed. The patterns of *Ctsk*, *Sp7*, *Col1a1* and *Dspp* gene expression were displayed. c) Cryostat section of *Ctsk‐GFP* mice molar at P1.5, P7.5, P14.5, 1 month, 18 month old. Arrows indicated Ctsk‐GFP^+^ cells and dotted line depicted vasculature(v). d) Quantification indicated the percentage of *Ctsk‐GFP^+^* cells versus DAPI^+^ cell within crown odontoblasts and crown middle papilla or pulp, respectively (n = 6 mice). Scale bar:50 µm.

Dental pulp continuously forms dentin throughout the entire life process, from developing primary dentin before tooth eruption to secondary dentin after tooth eruption by a gradually weakening dentin formation speed. Our first attempt to explore dental pulp lineage was to retrieve and analyze publicly accessible single‐cell sequencing (scRNA‐seq) datasets of neonatal mouse molar. Pulp cells at postnatal day 7.5 (P7.5) were divided into five clusters: mesenchymal, endothelial, perivascular, immune, and epithelial cells.^[^ ^20^ ^]^ We found that *Ctsk* was mainly expressed within the mesenchymal cell cluster (**Figure** **1**a). Mesenchymal cells could be further divided into coronal, middle, and apical papilla cells, in addition to odontoblasts and follicle cells.^[^ ^20^ ^]^ *Ctsk* was expressed in all the above clusters of mesenchymal cells in a scattered pattern at P7.5 (Figure 1b). This is partially overlapped with classical odontoblast markers, including Sp7 transcription factor (*Sp7)*,^[^ ^21^ ^]^ collagen type I alpha 1 chain *(Col1a1)* ^[^ ^21^ ^]^ and dentin sialophosphoprotein (*Dspp)* ^[^ ^22^ ^]^ (Figure 1b).

To visualize the precise pattern of *Ctsk*‐expressing cells in vivo from developmental to aging odontogenesis, we generated *Ctsk‐H2BGFP* (*Ctsk‐GFP*) knock‐in reporter mice expressing nuclear green fluorescent protein (GFP) in Ctsk^+^ cells (Figure S1a‐c, Supporting Information). At P1.5, the *Ctsk‐GFP* signal was initiated in the middle papilla (≈1.70%) and undetectable at odontoblasts (≈0.00%) (Figure 1c,d). At P7.5, a stage of primary dentin development, the *Ctsk‐GFP* signal was increased (≈91.04%) as odontoblasts and remain limited in the rest of the papilla (≈9.46%) (Figure 1c,d). A further look at cell proliferation and differentiation indicated that the more toward the coronal, the Ctsk^+^ cells were more differentiated as Ctsk‐GFP^+^/Col2.3‐BFP^+^ double positive cells, and the more toward the apical, the Ctsk^+^ cell were more proliferative as Ctsk‐GFP^+^/ ki67 ^+^ double positive cells (Figure S1d, Supporting Information). At P14.5, a stage of crown thickening and root elongation, the *Ctsk‐GFP* signal was strong in the crown odontoblasts(≈91.93%), root pulp, and root odontoblasts, while remain limited in the rested crown pulp(≈7.99%) (Figure 1c,d). At 1 month, a stage of crown accomplishment and root thickening, the *Ctsk‐GFP* signal was dropped in the crown odontoblasts (≈48.79%), especially rare at the cusp, remain limited in the rested crown pulp (≈6.72%), while still remained strong in root odontoblasts(Figure 1c,d). By 18 months, a stage of aging, the number of *Ctsk‐GFP* signals were limited at odontoblasts(≈4.06%) and restricted to the vasculature within the rested pulp (≈10.22%) and apex (Figure 1c,d). A glance at statistical analysis indicated that *Ctsk‐GFP* signals at odontoblasts is associated with neonatal odontogenesis, while *Ctsk‐GFP* signals within the rest crown pulp is limited and stable chronologically, function of which is yet to be explored.

### 2.2 The Ctsk^+^ Lineage Contributes to Odontoblast and Dentin Formation

**Figure 2:**
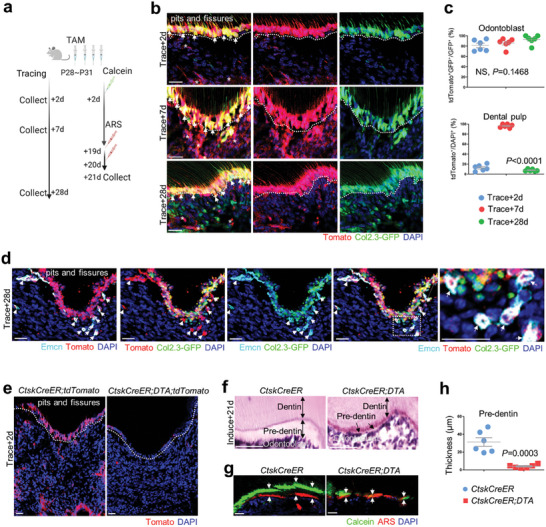
Adolescence Ctsk^+^ lineage contributes to dentin renewal. a) Schematics of experiments design. b) Lineage tracing using *Ctsk‐CreER;tdTomato;Col2.3‐GFP* mice. Odontoblasts were indicated by dotted line, double positive odontoblasts were indicated by arrows, labeled pulp cells were indicated by asterisk. c) Quantification indicated the percentage of *tdTomato^+^/Col2.3‐GFP^+^* cells versus GFP^+^ cell within odontoblasts, and the percentage of *tdTomaoto^+^* cells versus DAPI^+^ cells within the dental pulp areas of interest (n = 6 mice). d) Immunostaining of vasculature using Emcn antibody and merged respectively as indicated (arrows). e) Efficiency of Ctsk lineage depletion indicated by *tdTomato*. f) H&E staining indicated pre‐dentin formation (red dotted line). g) Double labeling using Calcein and Alizarin red indicated new dentin formation (arrow). Scale bar, 20 µm. h) Quantification of pre‐dentin thickness (n = 6 fields from four mice). Data are shown as the mean ± S.E.M, one‐way ANOVA with Tukey's post‐test (d), unpaired two‐tailed Student's t‐test i).

The pattern of *Ctsk*‐GFP^+^ cells led us to determine whether Ctsk^+^ lineage contributes to odontoblasts. Subsequently, we set up and induced *Ctsk‐CreER;tdTomato;Col2.3‐GFP* mice to trace Ctsk^+^ lineage from P28^[^ ^23^ ^]^ during mice adolescence (**Figure** **2**a). At odontoblasts layer, tdTomato^+^ cells were predominantly colocalized with *Col2.3‐GFP* ^+^ odontoblasts from two‐day tracing (≈81.21%), seven‐day tracing(≈85.85%) to 4‐week tracing(≈92.79%), (Figure 2b,c). Within dental pulp, tdTomato^+^ cells began with a sporadic distribution(≈12.66%), then increased to a predominate pattern(≈96.30%), and finally dropped back to the sporadic distribution(≈7.74%) (Figure 2b,c), where *tdTomato* ^+^ label‐retaining cells colocalized with the Emcn^+^ vasculature wall, suggesting a perivascular niche^[^ ^24^ ^]^ of Ctsk^+^ progenitors (Figure 2d). To overcome the potential limitation of inducible Cre, we also checked *Ctsk‐Cre;tdTomato;Col2.3‐GFP* mice as a supplementary tracing strategy. tdTomato‐labelled Ctsk^+^ lineage was also predominantly enriched with a close relationship with Osx^+^ and Col2.3GFP^+^ odontoblasts (Figure S2a, Supporting Information). Specifically at the odontoblast boundaries labelled by Col2.3‐GFP, Ctsk^+^ lineage surrounded Emcn^+^ vessels with a close relationship with β3‐tublin^+^ nerve fibres (Figure S2b, Supporting Information). Contribution to odontoblasts and label‐retaining cells indicate the dual identity of Ctsk^+^ lineage, suggesting its role for odontogenesis.

To confirm the role of Ctsk^+^ lineage for odontogenesis is indispensable, we generated *Ctsk‐CreER;tdTomato;DTA* mice to induce depletion of Ctsk^+^ lineage from P28 (Figure 2a). First, we confirmed the high efficiency of Ctsk^+^ lineage depletion, as indicated by the absence of tdTomato labelling in mutant molars (Figure 2e). Twenty‐one days after Ctsk^+^ lineage depletion, haematoxylin and eosin (H&E) staining indicated that pre‐dentin formation had terminated (Figure 2f,h). Calcein‐alizarin red double labelling indicated that dynamic dentin deposition was discontinued in the Ctsk^+^ lineage‐depleted molars when compared to the normal dentin formation rate (Figure 2g). These observations indicate that Ctsk^+^ lineage contributes to odontogenesis of mouse molar pulp.

### 2.3 Inferior Alveolar Nerve Denervation Halts Ctsk^+^ Lineage‐Supported Secondary Dentin Formation via Hedgehog Signalling

**Figure 3:**
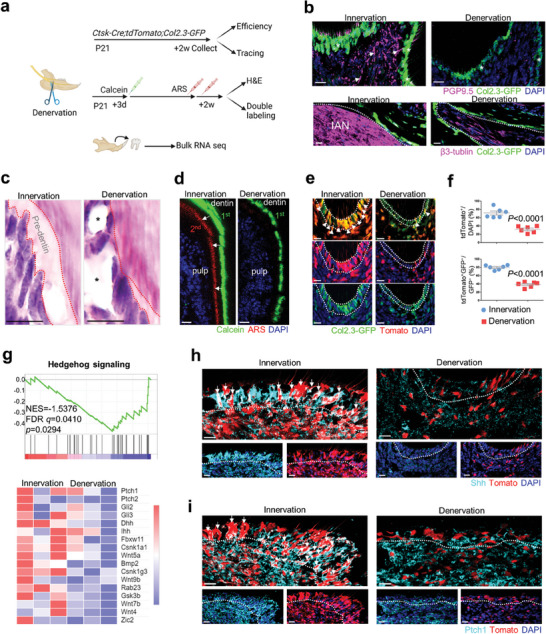
Denervation impaired odontogenesis and downregulated Hedgehog signaling. a) Schematics of experiments design. b) Immunofluorescent staining of PGP9.5 and βIII‐tublin antibody on *Col2.3‐GFP* mice. c) Enlarged view of H&E staining indicated impaired pre‐dentin formation (red dotted line) and enlarged vacuole (asterisk) within pulp tissue. Scale bar, 5 µm. d) Double labeling showed halted mineralization indicated by absent capture of Alizarin red line. e) Lineage tracing indicated decreased Ctsk^+^ lineage‐supported odontoblasts formation(arrow) on *Ctsk‐Cre;tdTomato;Col2.3‐GFP* mice. f) Quantification of *tdTomato^+^* cells versus DAPI^+^ cells and double positive cells versus GFP^+^ cells (n = 6 fields from four mice). Scale bar, 20 µm. g) GSEA showing decreased enrichment of Hh signaling pathway‐regulated gene expression and partial marker genes were indicated in the heatmap. h,i) Immunofluorescent staining using Shh and Ptch1 antibody respectively on molar pulp of *CtskCre;tdTomato;Col2.3‐GFP* mice. Dotted line indicated odontoblasts layer. Scale bar, 20 µm. Data are shown as the mean ± S.E.M, unpaired two‐tailed Student's t‐test (f).

Dental pulp is highly neurogenic,^[^ ^25^, ^26^ ^]^ leading us to raise a hypothesis that innervation supports molar odontogenesis by Ctsk^+^ lineage. To this end, we established a mandibular denervation mouse model via IAN transection surgical procedures to examine dentin histology, Ctsk^+^ lineage phenotype, and signalling analysis (**Figure** **3**a). We first confirmed IAN denervation indeed reduced PGP9.5^+^ nerve fibres within the inferior alveolar canal and molar pulp the inferior alveolar canal (Figure 3b). We found *Col2.3‐GFP* ^+^ odontoblasts became atrophic and reduced in number (Figure 3b). We observed enlarged vacuoles within pulp and reduced thickness of pre‐dentin in denervated group by H&E staining (Figure 3c; Figure S3a,b, Supporting Information). We also observed that stagnant dentin formation rate shown by the absence of Alizarin Red (ARS) (Figure 3d). These results indicates that loss of nerve halts pre‐dentin renewal and mineralization process during molar odontogenesis.

IAN is the sole sensory nerve,^[^ ^27^, ^28^ ^]^ leading us to tested next whether the loss of nociceptive nerve weakened Ctsk^+^ cells. In denervation model, we observed the number of tdTomato‐labelled Ctsk^+^ lineages and tdTomato/Col2.3‐GFP double‐positive cells decreased, morphologically which became shortened and atrophic (Figure 3e,f). IAN secretes calcitonin gene‐related peptide (CGRP), a marker for nociceptive nerve,^[^ ^27^, ^28^ ^]^ which is encoded by the *Calca* gene. To label nociceptive nerve and Ctsk^+^ cells simultaneously, we established *Calca‐Cre;tdTomato;Ctsk‐GFP* mice. The successful labelling of the nociceptive nerve is confirmed by tdTomato^+^ cells within IAN. We observed a consistently close relationship of tdTomato^+^ nociceptive nerve fibers with Ctsk‐GFP^+^ cells (Figure S3c, Supporting Information). Next, we ablated nociceptive nerve‐related cells by *Calca‐Cre;DTA;Ctsk‐GFP* mice. We found decreased primary dentin thickening by quick xylenol orange (XO) staining and reduced number of Ctsk‐GFP^+^ cells, whereas the non‐ablation group showed normal crown dentin thickness with tubules and Ctsk‐GFP^+^ functioning odontoblasts (Figure S3d, Supporting Information). These results indicated that nociceptive nerve promotes dentin thickening supported by Ctsk^+^ cells.

To further test whether Ctsk ^+^ cells‐supported odontogenesis requires nerve‐related signalling, we did bulk RNA‐seq analysis of denervated mouse molar pulp tissue (Figure 3a). We first confirm the downregulation of axon development‐ and extension‐related genes by gene set enrichment analysis (GSEA), indicating successful establishment of IAN denervation again (Figure S3e, Supporting Information). We found Hh signalling pathway‐related genes were downregulated, and the heatmap showed part of downregulated genes related to Hh signalling (Figure 3g). IAN synthesizes and secrete Shh,^[^ ^24^ ^]^ an Hh ligand, binds to the membrane receptor Patched 1 (Ptch1)^[^ ^29^, ^30^ ^]^ and activates the Hh signalling pathway, which is crucial for skeletal development and regeneration.^[^ ^31^, ^32^ ^]^ Immunostaining showed that Shh ligand binding to the tdTomato‐labelled Ctsk^+^ lineage decreased after denervation (Figure 3h). Ptch1 expression in tdTomato‐labelled Ctsk^+^ cells also decreased after denervation, indicating reduced Hh signalling (Figure 3i). These results indicated that loss of nociceptive nerve weakens Ctsk ^+^ cells for odontogenesis somehow by downregulating Hh signalling.

### 2.4 Loss of Nerve‐Derived Shh Impairs Ctsk^+^ Cell‐Supported Odontogenesis

**Figure 4:**
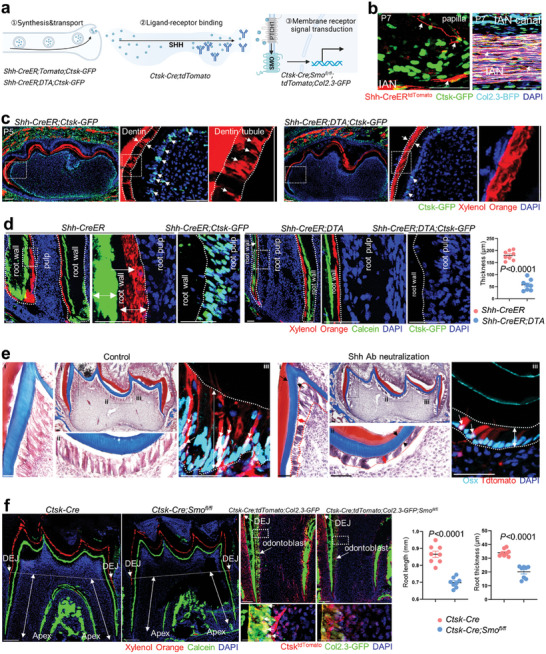
Loss of Shh signaling suppressed neonatal odontogenesis. a) Schematics of blocking Shh signaling by ① synthesis and transport, ②ligand‐receptor binding and ③membrane receptor‐mediated signal transduction. b) Cryostat section of *Shh‐CreER;tdTomato;Ctsk‐GFP* mice molar at P7, Tomato‐labeled Shh‐secreting nerve fibers grow into papilla and pass through IAN canal indicated by arrows. c) Cryostat section of *Shh‐CreER;DTA;Ctsk‐GFP* mice molar at P5, reduced crown dentin(dotted line) and Ctsk‐GFP^+^ cells (arrow) were indicated. d) Double labeling of *Shh‐CreER;DTA* mice molar at P21, reduced labeling of xylenol orange and calcein (dotted line) and Ctsk‐GFP^+^ cells (arrow) of *Shh‐CreER;DTA;Ctsk‐GFP* mice were indicated. Quantification of root dentin wall thickness was shown aside (n = 8 fields from six mice). e) Masson trichrome staining indicated suppressed odontoblasts (dotted line) and reduced dentin thickness (arrows) after Shh neutralization. Framed areas were applied with immunostaining analysis of Osx antibody and enlarged besides. f) Double labeling of *Ctsk‐Cre;Smo^fl/fl^* mice and tracing of *Ctsk‐Cre;Smo^fl/fl^;tdTomato;Col2.3‐GFP* mice molar at P19. Dentin‐enamel‐joint(DEJ), apex, root length(double arrow) and reduced odontoblasts(enlarged, arrow)were indicated. Quantification of root length and root wall thickness were indicated aside (n = 8 fields from six mice). Data are shown as the mean ± S.E.M, unpaired two‐tailed Student's t‐test (d,f). Scale bar, 50 µm.

On the basis of our understanding of Hh signalling involved in Ctsk^+^ cells‐supported odontogenesis, we next asked whether our observed Shh expression was correlated during physiological dentin formation. To this end, we blocked Shh signalling through Shh synthesis, ligand‐receptor binding, and membrane receptors, respectively (**Figure** **4**a; Figure S4a, Supporting Information).

Aiming at the upstream, we first labelled the Shh‐synthesis lineage using *Shh‐CreER;tdTomato;Ctsk‐GFP;Col2.3‐BFP* mice induced from P1.5 and harvested at P7.5. We confirmed the successful labelling of the tdTomato^+^ axons through the Col2.3‐BFP^+^ IAN canal(Figure 4b). We found *Shh‐CreER^tdTomato+^* axons reached Ctsk‐GFP cells in the dental papilla at P7.5, thus constituting a major source Shh protein (Figure 4b). Next, we ablated the Shh‐secreting lineage using the DTA allele from P1.5 and harvested the P5.5. Reduced Shh and Ptch1 protein expression colocalized with Ctsk‐GFP validated that Shh signalling was downregulated within Ctsk^+^ cells (Figure S4b, Supporting Information). We found the reduced thickness of dentin and loss of normal dentin tubules by quick xylenol orange staining, reduced number of Ctsk^+^ cells in ablated groups (Figure 4c). To rule out the effect of Shh secreted from ameloblasts, we abated the Shh‐secreting lineage from P21 to P28 during the root thickening stage after ameloblast differentiation was finished. We observed almost stagnant root dentin formation with absence of Ctsk^+^ cells in the ablated groups, the thickness of which was around one‐third of that in the non‐ablated group with abundant Ctsk^+^ cells (Figure 4d). These results indicated that nerve‐derived Shh promotes Ctsk^+^ cells for dentin thickening.

To interfere cells from Shh ligands binding, we injected Shh neutralisation antibody into *Ctsk‐Cre;tdTomato* mouse pups. We confirmed a high efficiency of neutralisation via immunostaining with Shh antibody, which is merged with reduced number of tdTomato‐labelled Ctsk^+^ lineage (Figure S4c,d, Supporting Information). We observed that primary dentin thickness was reduced at P8.5 by Masson's trichrome (Figure 4e; Figure S4e, Supporting Information). By serial section at aside, we found the reduced number of Osx^+^/tdTomato^+^ double‐positive Ctsk^+^ lineage, and the reduced cellular/ nuclear height (Figure 4e; Figure S4e,f, Supporting Information). These results indicated that interfered Shh ligands binding process weakens Ctsk^+^ lineage‐contributed odontogenesis during primary dentin formation.

Furthermore, we rationalized that if Shh ligands directly bind to Ctsk lineage, then genomic deletion of Smo membrane receptor by *Ctsk‐Cre* will also reduce odontogenesis. We therefore set up *Ctsk‐Cre;Smo^fl/fl^;tdTomato;Col2.3‐GFP* mice and conducted double labelling from P10 to P19, a stage of robust odontogenesis for root elongation. In low magnification, we found the root elongation was decreased from the dentin‐enamel junction (DEJ) to the apex by ≈19.33%, and the root wall thickness was also decreased by ≈40.70% (Figure 4f). In higher magnification merged with double labelling, tdTomato^+^/Col2.3‐GFP ^+^Ctsk^+^ lineage was weakened at the inner surface of the root wall (Figure 4f). These results indicated that Ctsk^+^ lineage requires nerve‐originated Shh to contribute odontogenesis during primary odontogenesis.

### 2.5 Shh is Required for Ctsk^+^ Cell‐Mediated Reactionary Dentin Formation During Injury Repair

**Figure 5:**
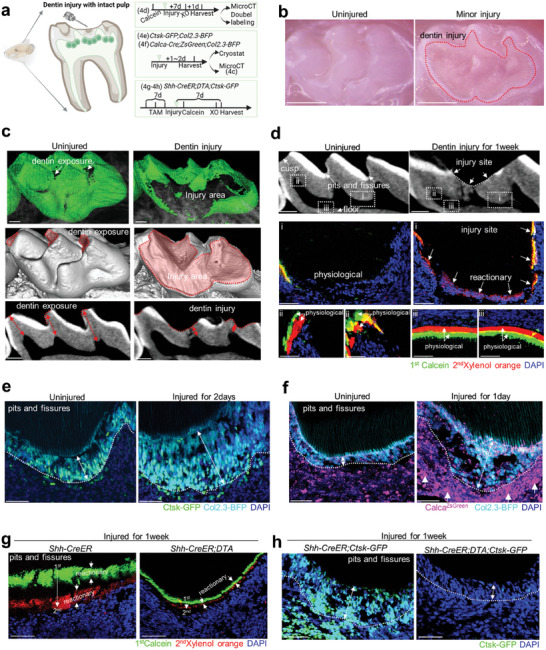
Dentin injury enhanced *Ctsk‐GFP* ^+^ cells and dentin regeneration. a) Schematics of dentin injury mouse molar model. b) Stereoscopic view of dentin injury (dotted line) at occlusal site of mouse molar at age of 6 weeks. c) 3D reconstruction of enamel (upper lane, green), whole crown (middle lane,) and sagittal view of the crown (lower lane) of detin injury mouse molars. The affected dentin area (dotted line) and corresponding dentin thickness (double arrow) was indicated in control and injured group. d) Double labeling of mouse molars seven days after the dentin injury. Pulp areas beneath pits and fissures (i), cusp (ii), and floor(iii) are framed and enlarged. Reactionary dentin formation is indicated by arrows in enlarge pulp area of pits and fissures. e) Cryostat section of *Calca‐Cre;ZsGreen;Col2.3‐BFP* mice molar 24 h after the dentin injury. *Col2.3‐BFP* ^+^ elongated odontoblasts(dotted line, double arrow) and increased nociceptive nerve fibers(colorized by magenta, arrow) were indicated. f) Cryostat section of *Ctsk‐GFP;Col2.3‐BFP* mice molar 48 h after the dentin injury. *Ctsk‐GFP^+^Col2.3‐BFP* ^+^ elongated odontoblasts(dotted line, double arrow) were indicated. g) Double labeling of *Shh‐CreER;DTA* mice molar 7 days after the dentin injury. First calcein was injected one days after the dentin injury and second xylenol orange 1 day before harvest 7 days after the injury. h) Cryostat section of *Shh‐CreER;DTA;Ctsk‐GFP* mice molar seven days after the dentin injury. *Ctsk‐GFP^+^* odontoblasts (dotted line, double arrow) were indicated. Scale bar, 50 um.

As dental pulp has the self‐repairing capability but in an extremely limited manner, we hypothesized that Shh may play a critical role activating Ctsk^+^ cell‐mediated reactionary dentin formation in this turning point before irreversible pulpitis. We first set up a mouse dentin injury model by drilling molar dentin with minor injury without damaging the pulp (**Figure** **5**a,b). We reconstructed the 3D view of injured molar and measured that the affected dentin thickness remained ≈0.05 mm, while the uninjured dentin ≈0.15 mm (Figure 5c; Figure S5a, Supporting Information). The reactionary dentin only forms beneath the injury site, which is distinctive from secondary dentin. We conducted double labelling and found our injury stimulated newly‐formed mineralization at pits and fissures, while secondary dentin is comparable at cusp/floor in contrast to the uninjured (Figure 5d). These results indicated that we successfully simulate clinical situation of dentin injury with reactionary dentin formation.

To explore how Ctsk^+^ cell is associated, we set up the dentin injury model on *Ctsk‐GFP;Col2.3‐BFP* mice. At the first two days after the injury, we observed the height of Ctsk‐GFP^+^/Col2.3‐BFP^+^ double positive odontoblasts was around two to three times than the uninjured (Figure 5e,f; Figure S5b,c, Supporting Information). The number of Ctsk‐GFP^+^ cells per area was around four times greater than the uninjured (Figure 5e; Figure S5b, Supporting Information). The number of Calca^+^ nociceptive nerve fibres was around two times greater than the uninjured (Figure 5f; Figure S5c, Supporting Information). One week after the injury, tdTomato^+^/Col2.3‐GFP^+^ double positive cells beneath the injured site remained modestly elongated (Figure S5d, Supporting Information), with increased CGRP^+^ nerve fibres ascending (Figure S5e,f, Supporting Information). Emcn^+^ vessels were in close relationship beneath the uninjured Ctsk^+^ lineage, while increased but limited when mesial cusp was injured restrictedly (Figure S5g, Supporting Information). These data indicate that Ctsk^+^ cells are required for secondary dentin formation due to minor injury, which is associate with CGRP^+^ nerve and Emcn^+^ vessels.

To test whether Shh is required for reactionary odontogenesis, we ablated the Shh‐secreting lineage using the DTA allele at adolescence, set up dentin injury model and conducted double labelling experiment (Figure 5a). We found the reactionary mineralization was weakened, and dentin formation rate were almost ceased (Figure 5g). By serial section, we also observed that Ctsk^+^ cells was almost eliminated and the height of odontoblasts was reduced, compared to those within the injured *Shh‐CreER;Ctsk‐GFP* mice (Figure 5h). These data indicate that nerve‐derived Shh promotes Ctsk^+^ cells contributing to reactionary odontogenesis due to minor injury to the mouse molar. This result brings up an interesting possibility that Shh may affect the range of pulp's capabilities of self‐repairing.

### 2.6 Shh Expands the Limit of the Self‐Repairing Capability by Promoting Ctsk^+^ Cell Vitality in Crown Pulp Exposure

**Figure 6:**
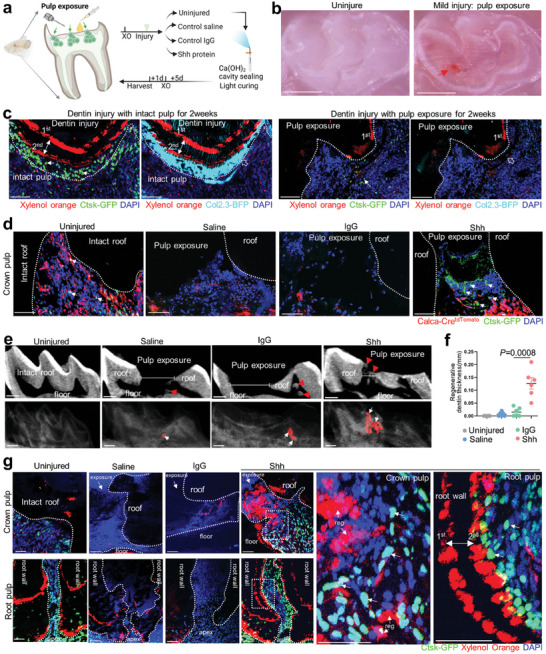
Shh protein to pulp exposure enhanced *Ctsk‐GFP^+^* cells and dentin regeneration. a) Schematics of Shh capping to pulp exposure mouse molar model. b) Stereoscopic view at occlusal site of mouse molar immediately after pulp exposure (arrow) at age of 6 weeks. c) Comparison of *Ctsk‐GFP^+^* cells(arrow), *Col2.3‐BFP* ^+^ odontoblasts (hollow arrow) and double labeling of xylenol (double arrow) between dentin injury and pulp exposure group two weeks after the injury. d) Cryosection of *Calca‐Cre;tdTomato;Ctsk‐GFP* mice molar at two days after pulp exposure in uninjured group, saline group, IgG group and Shh group. Tomato‐labeled nociceptive associated cells and Ctsk‐GFP^+^ cells were indicated by arrows. e,f) 2D sagittal view of the injured molar (defects width was indicated) and 3D reconstruction of reparative dentin (arrow) in uninjured group, saline group, IgG group and Shh group. Quantification of regenerative dentin thickness. (n = 6 fields from four mice). g) Undecalcified frozen slides of regenerative dentin (double labeling of xylenol orange, double arrow, reg) and *Ctsk‐GFP* ^+^ cells(arrow) on exposed crown pulp and corresponding apex in uninjured group, saline group, IgG group and Shh group (e). Areas of Shh‐preserved crown pulp and root pulp are enlarged aside. Data are shown as the mean ± S.E.M, one‐way ANOVA with Tukey's post‐test. Scale bar, 50 um.

Next, we hypothesized that Shh may promote regenerative capability of affected pulp when resisting mild injury. We first established a pulp exposure mouse model addressing the clinical aim of crown pulp vitality preservation (**Figure** **6**a,b). We confirmed the determining feature of pulp exposure model was irreversible loss of Ctsk‐GFP^+^ cells and Col2.3‐BFP^+^ odontoblasts, and ceased dentin formation rate, in contrast to Ctsk^+^ cells‐supported odontogenesis during dentin injury (Figure 6c). To test the hypothesis, we directly and precisely capped Shh protein to the exposed pulp horn under microscopy. At neuro‐cellular level within crown pulp, the Shh‐capping group preserved Ctsk‐GFP^+^ cells associated with Calca^+^ nociceptive nerve‐related cells, similar to the uninjured group two days after pulp exposure, while the other groups without Shh showed dying Ctsk‐GFP and lost tdTomato signals (Figure 6d). At dentin mineralization level of injured crown, Shh‐capping induced mineralization seeming to fix the exposure, increasing the preserved dentin volume and the resistance to occlusal force (Figure 6e,f). In contrast, capping without Shh almost ceased mineralization and the dentin defect was wider when gradually losing resistance to the occlusal force as irreversible pulpitis (Figure 6e,f). At Ctsk‐GFP^+^ cells‐supported odontogenesis level of crown and root, the Shh‐induced reparative dentin is supported by Ctsk‐GFP^+^ cells, whereas the saline and IgG groups hardly formed pulp calcification or Ctsk^+^ cells (Figure 6g). Looking further into subsequential root thickening at cellular level, we explicated that root dentin thickening was preserved by Shh‐capping and supported by Ctsk‐GFP^+^ cells, resembling physiological status, whereas the saline and IgG groups showed no new root wall formation or living Ctsk^+^ cells surrounding them (Figure 6g). Our data indicated that Shh, via direct contact with the crown exposed pulp, could expand the limit of reparative dentin formation capability by enhancing Ctsk^+^ cells.

### 2.7 Shh Promotes Apex Odontogenesis when Root Pulp is Affected for Young Molars

**Figure 7:**
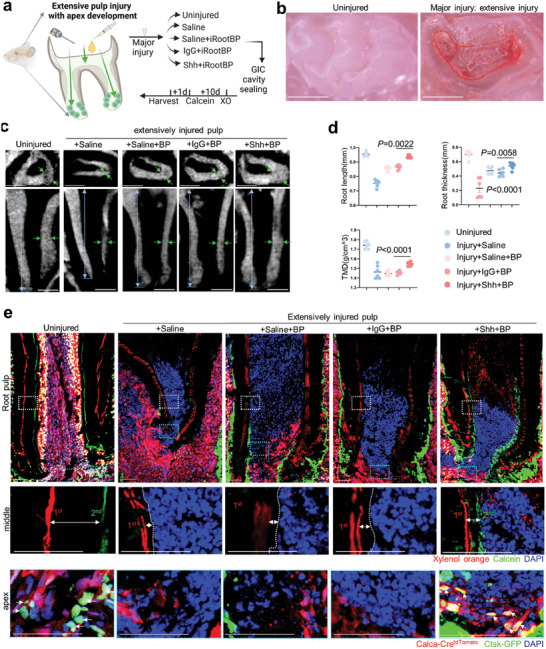
Shh protein to injured crown pulp preserved apex *Ctsk‐GFP^+^* cells and root dentin formation. a) Schematics of Shh protein administration to extensive pulp injury mouse model. b) Stereoscopic view of crown pulp injury (dotted line) at occlusal site of mouse molar at age of 3–4weeks. c,d) 2D sagittal view of developing palate root after crown pulp injury in uninjured group, saline group, saline+BP group, IgG+BP group and Shh+BP group. Arrows indicated thickness and length of growing root. Quantification of root length, TMD and root wall thickness in c. e) Undecalcified frozen slides of growing root of *Calca‐Cre;tdTomato;Ctsk‐GFP* mice molar after crown pulp injury in above five groups. Double labeling of xylenol orange and calcein (double arrow) and *Ctsk‐GFP* ^+^ cells associated or merged with tomato (arrow) were enlarged. Exposed crown pulp and corresponding apex in saline group, IgG group and Shh group (e). Areas of Shh‐preserved crown pulp and root pulp are enlarged aside. Quantification of regenerative dentin thickness f). (n = 6 fields from four mice). Data are shown as the mean ± S.E.M, one‐way ANOVA with Tukey's post‐test (d). Scale bar, 50 um.

As root canal infection hinders apex development, we then asked whether Shh could promote root odontogenesis when pulp inflammation proceeds in depth by major injury. To this end, we established the extensive pulp injury model to simulate the clinical situation of apex development for infected young permanent tooth (**Figure** **7**a,b). We directly sealed Shh protein to the injured crown pulp. Compared to clinically positive controls, root dentin formation was enhanced by an increase of ≈7.17% in root length, ≈6.18% in tissue mineralisation density (TMD), and ≈22.15% in thickness (Figure 7c,d). Compared to the physiological development of uninjured group, the apex of Shh group was less developed with a decrease of ≈1.83% in root length, ≈11.28% in TMD, and ≈22.65% in thickness (Figure 7c,d). These data indicated that Shh protein sealing to the injured crown pulp promote odontogenesis of root development for adolescence to some extent even if the inflammation remains.

Furthermore, we asked whether the neuro‐cellular pulp reaction was regulated by the external Shh. We perform the extensive pulp injury and sealed Shh protein to the *Calca‐Cre;tdTomato;Ctsk‐GFP* mice. We found the apical dentin formation was still ongoing by nice uptake of two dosage of double labelling (Figure 7e). By magnified view of apical area, we observed Ctsk^+^ cells merging with the tdTomato^+^ nociceptive nerve‐related cells were still preserved, while the root pulp was irreversibly necrotic (Figure 7e). The groups without Shh‐sealing exhibited varying degrees of pulpitis or pulp necrosis with stagnant root dentin formation, indicated by the absence second uptake of calcein and the loss of Ctsk^+^ cells and tdTomato^+^ cells (Figure 7e). Our data indicated that exogenous Shh preserved the apical odontogenesis capability by activating Ctsk^+^ cells to some extent, while still could not terminate the progression of pulpitis or pulp necrosis.

### 2.8 Shh Activates aged Ctsk^+^ Cells Surrounding the Vasculature Responding to Dentin Injury Repair

**Figure 8:**
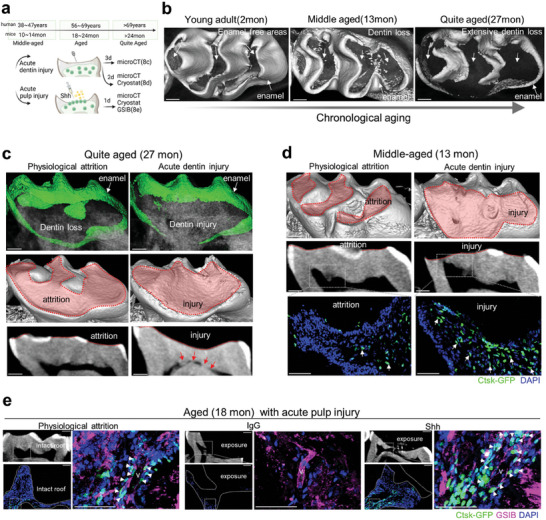
Aged *Ctsk‐GFP* ^+^ cells around vasculature were still responsive to Shh protein when injured. a) Schematic of the experiments design. a 3D reconstruction of enamel (white arrow) and dentin exposure area(red) of mouse molars at the age of 2 months, 13 months and 27 months. b) 3D reconstruction of enamel (colorized green) and whole crown(attrition or injury area by dotted line), and 2D sagittal view of crown of uninjured and injury mouse molars at the age of 27 months. Reactionary dentin three days after the injury was indicated by red arrows. c) 3D reconstruction of whole crown(attrition or injury area by dotted line), and 2D sagittal view of crown of uninjured and injury mouse molars at the age of 13 months. Reactionary *Ctsk‐GFP* ^+^ cells 2 days after the injury was indicated by arrows. d) 2D sagittal view of crown of uninjured group, IgG group and Shh group of Ctsk‐GFP mice at the age of 18 months. Staining of GSIB (v: vasculature, recolored as magenta) on *Ctsk‐GFP* ^+^ cells(arrows) one days.Scale bar, 50 um.

Chronological aging is regarded as another form of inflammation and promotes chronical dentin injury.^[^ ^33^ ^]^ In previous clinical efforts to age‐related pulp treatment, the external capping for the purpose of vital pulp preservation is often invalid, leading us to ask whether Shh could bring us possibilities of pulp preservation for aged molars. According to age equivalence between human and mice,^[^ ^34^, ^35^ ^]^ we set up chronological aging mouse model of adult group (mice below 6 months is equivalent to human under 30 years old), middle aged group (mice from 10 to 14 month old to human 38 to 47years old), and aged group (mice from 18 to 24 month old to human 56 to 69 years old) (**Figure** **8**a). Considering the increasing life expectancy to 73.4 years in 2019 estimated by the Global Health Observatory,^[^ ^36^, ^37^ ^]^ we set our end point of mice aging at 27 months old as quite aged group. We assessed the model of chronological aging molar was successful by 3D reconstruction and ensure the pulp was still intact (Figure 8b).

We asked whether the aged pulp still hold the endogenous capacity to form reactionary dentin. To this end, we acutely injured the quite aged mouse molar dentin, not reaching the pulp, without causing pulpitis nor apical periodontitis (Figure 8c; Figure S6a, Supporting Information). Compared to the age‐related narrow pulp chamber of the uninjured molars, the injured aged mouse molars showed rapid reactionary dentin formation beneath the injury site within 3 days of observation (Figure 8c; Figure S6b, Supporting Information).

Next, to test the aged Ctsk^+^ cells still support reactionary dentin formation, we injured the dentin without reaching the pulp of Ctsk‐GFP mouse molars at 13 months old for 2 days. Dentin injury promoted the number of Ctsk^+^ cells beneath the injury site, which was four times greater than uninjured Ctsk^+^ cells in a scattered pattern in the middle pulp area (Figure 8d; Figure S6c, Supporting Information).

Furthermore, to test the aged Ctsk^+^ cells could still be activated by exogenous Shh protein, we applied Shh protein and IgG as a control respectively to the injured pulp of Ctsk‐GFP mouse molars at 18 months old for 1 day. In uninjured aged Ctsk‐GFP mouse molars, dispersed Ctsk^+^ cells surrounded the vasculature labelled by GSIB (Figure 8e; Figure S6d, Supporting Information). In pulp‐injured aged Ctsk‐GFP mouse molars, the IgG group showed vascular ingrowth but lacking Ctsk^+^ cells. Shh group showed an increased number of Ctsk^+^ cells surrounding the vasculature, which is six times greater than uninjured group. Our data indicate that the aged mouse molar pulp reserves the endogenous odontogenesis capacity supported by Ctsk^+^ cells surrounding vasculature, which could be re‐activated by exogenous Shh administration.

## 3 Discussion

Due to dental pulp heterogeneity, targeting an essential lineage in molars is required, especially for economical and highly effective clinical treatment based on vital pulp preservation. The odontoblast line at the pulp‐dentin boundary originates from ecto‐mesenchymal cells, which contribute to post‐injury repair,^[^ ^38^, ^39^ ^]^ the apical papilla retains bipotent progenitor cells.^[^ ^20^, ^40^ ^]^ Mouse incisors possess niches for slow‐cycling cells that express GLI family zinc finger 1 (Gli1),^[^ ^24^ ^]^ proteolipid protein 1 (PLP1),^[^ ^41^ ^]^ and Thy‐1 cell surface antigen (Thy1),^[^ ^42^ ^]^ transit amplifying cells (TACs) expressing platelet‐derived growth factor receptor beta (PDGFRβ)^[^ ^43^ ^]^ and Axin2,^[^ ^44^ ^]^ as well as nerve/glial antigen 2 (NG2)‐expressing pericytes.^[^ ^45^ ^]^ Mouse molars contain mesenchymal progenitor cells that express osterix (Osx)^[^ ^21^, ^46^ ^]^ and paired related homeobox‐1 (Prx1),^[^ ^47^ ^]^ in addition to pericytes positive for *α*‐smooth muscle actin (*α*SMA).^[^ ^48^, ^49^, ^50^ ^]^ Most related lineage‐tracing assays have been performed in incisor mouse models owing to their lifelong continuous growth, while the more static molars bear a stronger resemblance to human teeth. The latest single‐cell atlas of mouse molars revealed a dynamic odontogenic population arising from diverse developmental stages.^[^ ^20^ ^]^ Our work revealed that a lifelong Ctsk‐expressing lineage in mouse molars is required for odontogenesis and is regulated by nociceptive nerve. Ctsk‐expressing lineage is presumably a suitable target lineage for cell‐based therapeutic approaches to vital pulp preservation (Figure S7, Supporting Information).

Acute pulp disease is associated with pain, which could be double‐edged and how to take advantages of the “pain” is somehow a mystery. The nociceptive nerves provide protection through communication and are more than a means of sensory conduction.^[^ ^51^ ^]^ In skin immunity, nociceptive sensory neurones detect external stimuli and activate local immune responses against pathogens.^[^ ^52^ ^]^ Nociceptive neurons mobilize haematopoietic stem cells (HSCs) in the bone marrow microenvironment.^[^ ^27^ ^]^ Free nerve endings of trigeminal neurons innervate the pulp‐dentin complex, which is highly specialized in external stimuli sensing and conducts pain in the form of an alert.^[^ ^53^ ^]^ By analyzing the turning point of dentin injury, we demonstrated that the molar nerve stimulates the Ctsk^+^ lineage to regain its odontogenic capability via Shh ligands. Further, we revealed that the nociceptor nerve not only serves as an alarm as self‐protection mechanism, but more importantly secrete Shh for dentin homeostasis and injury repair in mouse molars.

Shh signalling may be a hub pathway, which connects developmental odontogenesis to progressive injury repair. Shh^[^ ^54^, ^55^ ^]^ expression is crucial to the development of the central nervous system^[^ ^56^ ^]^ and neonatal calvaria.^[^ ^57^, ^58^ ^]^ Developmentally, our work underlines the role of shh to mice neonatal dentin formation. At this time the source of shh protein is IAN axons^[^ ^24^ ^]^ not epithelium.^[^ ^59^ ^]^ Injury triggers natural fracture healing in long bones,^[^ ^60^ ^]^ whereas dentin injury is less reversible. Skeletal stem cells were shown to respond to mechanical injury and activate bone regeneration by reverting to a developmental state in a jawbone injury mouse model.^[^ ^61^ ^]^ Our data confirmed that Ctsk^+^ cells are responsive to injury to initiate restorative dentin formation, which also regulated by nerve‐derived shh. Based on the dual role of shh in development and injury repair, we hypothesize that nerve‐derived shh may promote dentin formation for severe pulp injury and even aged pulp injury. A recent study also supports that mouse molar injury synergistically upregulates Shh and Cgrp expression.^[^ ^6^ ^]^ Our data indicated that shh protein strengthen the resistance of Ctsk^+^ cells to injury and promote odontogenesis from the perspective of age timeline and tooth disease progression, hopefully to be applied to a wilder field of clinical issues.

Overall, nerve‐regulated lifelong Ctsk^+^ cells are crucial for odontogenesis and Shh tilts the balance from pulp inflammation to dentin regeneration, providing valuable insights for therapeutic strategies designed to preserve pulp vitality.

## 4 Experimental Section

### Generation of Conditional *Ctsk‐H2BGFP* Mice

Four exons (exons 2–5) of the *Ctsk* gene, starting from the start codon ATG, were replaced with the coding region of H2B‐ eGFP and WPRE‐pA. A germline‐transmitted F1 founder was obtained by mating F0 generation‐positive mice with wild‐type mice according to the protocols for genotyping *Ctsk‐GFP* in Table S1 (Supporting Information).

### Mouse Study

All mouse experiments were approved by the Ethics Committee of the West China School of Stomatology, Sichuan University (WCHSIRB‐D‐2019‐062). *Ctsk‐Cre* mice were a generous gift from Dr Weiguo Zou, University of Chinese Academy of Sciences.^[^ ^15^ ^]^ *Ctsk‐CreER*, *Col2.3‐GFP*, *Col2.3‐BFP*,^[^ ^62^ ^]^ *Rosa26‐ZsGreen* ^[^ ^23^ ^]^ and *Rosa26‐DTA* mouse strains were kindly provided by Dr Bo O. Zhou, University of the Chinese Academy of Sciences. *Rosa26‐tdTomato* (007905) mice were obtained from Jackson Laboratory. *Calca‐Cre and Shh‐CreER* mice were purchased from Shanghai Model Organisms. *Smo^fl/lf^* mice were purchased from Cyagen Biosciences.

All the mice were housed under specific pathogen‐free conditions. Both male and female mice were analyzed. The mice were carefully euthanized using CO_2_. Genotypes were identified via PCR amplification of tail genomic DNA. *Ctsk‐CreERT2* was induced by tamoxifen (Sigma–Aldrich) dissolved in corn oil (Sigma–Aldrich, 20 mg mL^−1^) via daily intraperitoneal injection (200 mg kg^−1^) for four consecutive days.

### Denervation Mouse Models

Denervation surgery by inferior alveolar nerve transection was performed as per a previous protocol.^[^ ^63^, ^64^ ^]^ Briefly, P21 mice were anaesthetized with ketamine (100 mg kg^−1^) and xylazine (10 mg kg^−1^) and at least 2 mm of the IAN in the mandibular canal was transect.

### Dentin and Pulp Injury Mouse Models

It established three types of acute injury mouse models, the dentin injury model, pulp exposure model, and extensive pulp injury model, as follows:

- Dentin injury model with intact pulp: a low‐speed microscopic bur was used with cooling water and diluted a hole in the first maxillary molar of the mice at age of 6 weeks. The drilling depth reached the molar dentin without injuring the pulp horn.
- Pulp exposure model: A low‐speed microscopic bur with cooling water was used to gently dilute the first maxillary molars of the mice into the pulp horn at age of 6 weeks. Bleeding was stopped using sterile saline solution. Prior to routine calcium hydroxide pulp capping,^[^ ^65^ ^]^ 10 µL sterile saline or 100 µg mL^−1^ mouse Shh protein solution (464‐SH‐025/CF, R&D) were applied to the pulp tissue, followed by the application of a thin layer of light‐curing radiopaque calcium hydroxide paste (Calcimol LC) to avoid occlusal trauma. LC, a clinical reagent for pulp capping, was used as a positive control. Four groups were set up: the uninjured group (normal), pulp exposure group capped with saline (saline with LC), pulp exposure group capped with IgG (IgG with LC), and pulp exposure group capped with Shh (Shh with LC) before calcium hydroxide paste capping.
- Extensive pulp injury model: It established a mouse molar pulp injury model by completely removing the chamber roof and exposing the whole crown pulp at age of 3–4weeks. iRoot BP (BP) was used, the clinical reagent for pulp preservation, as positive control. It included five groups: the uninjured group (normal), dentin injury group sealed with saline only (saline without BP), dentin injury group sealed with saline before iRootBP capping (saline with BP), dentin injury group sealed with IgG before iRootBP capping (IgG with BP), and dentin injury group sealed with Shh before iRootBP capping (Shh with BP). The cavity was sealed with glass ionomer cement(GIC).

### Histological preparation and staining

The mandible and maxilla were cryostat‐sectioned into 10‐µm‐thick sections with a freezing microtome (CM3050S; Leica). H&E staining (Biosharp) and Masson's trichrome staining (ab150686; Abcam) were performed according to the manufacturer's instructions.

For immunofluorescence staining, slides were then incubated with the indicated antibodies at 4 °C overnight: Goat anti‐Endomucin (1:200, AF4666, R&D), Rabbit anti‐PGP 9.5 (1:200, ab108986, Abcam), Goat anti‐Patched / PTCH1 (1:200, ab109096, Abcam), Goat anti‐Sonic Hedgehog (1:200, ab240438, Abcam), Rabbit anti‐Sonic Hedgehog (1:200, ab53281, Abcam), Rabbit anti‐Ki67 (1:200, ab15580, Abcam), Rabbit anti‐Osterix (1:300, ab22552, Abcam), or Rabbit anti‐Ctsk (1:100, A1782, Abclonal). Sections were incubated with the indicated secondary antibodies at room temperature for 2 h: Donkey anti‐Goat Alexa Fluor 647 (1:200, ab150131, Abcam), Donkey anti‐Rabbit Alexa Fluor 647 (1:200, ab150067, Abcam). Sections were imaged using a laser‐scanning confocal microscope (FV3000; Olympus).

### Calcein‐Alizarin Red S Labelling

Calcein‐Alizarin Red S labelling was performed as previously described.^[^ ^66^ ^]^ Briefly, the mice were injected intraperitoneally with 20 mg kg^−1^ calcein (1 mg mL^−1^ in 2% NaHCO_3_ solution) and 40 mg kg^−1^ Alizarin Red S (2 mg mL^−1^ in H_2_O) as indicated in the schematics of each Figure Undecalcified specimens were cut into 10‐µm‐thick sections with a freezing microtome (CM3050 S; Leica) using an adhesive film system (Cryofilm; Section‐Lab Co., Ltd.).^[^ ^67^ ^]^

### Xylenol Orange Labelling

Xylenol orange labelling was performed on *Ctsk‐GFP* mice as previously described.^[^ ^68^, ^69^ ^]^ Briefly, mice were injected with 10 mg kg^−1^ xylenol orange (398187‐1G, Sigma) one day before harvest.

### Micro–Computed Tomography Imaging and Analysis

General parameters: The specimens were scanned with a µCT‐50 system (Scanco Medical) at a medium resolution and a voxel size of 5 µm. IMARIS software (version 9.1.2; Oxford Instruments) was used for data analysis and image processing.

3D‐reconstruction of aged mouse molars: Mouse molars were harvested at the age of 2 months (young adults), 13 months (middle‐aged), and 27 months (aged). By reconstructing the enamel of the mouse molar and re‐colouring as white or green, the condition of mouse molars were illustrated with aging.

### Bulk RNA‐seq Analysis

Total RNA from the dental pulp was isolated from the mandibular molars after removing the periodontal tissue using TRIzol reagent (Invitrogen), according to the manufacturer's protocol. Six molars with intact IAN (control) and six molars with IAN transection (denervation) were used for total RNA isolation.

The libraries were sequenced using the Illumina NovaSeq 6000 platform, and 150 bp paired‐end reads were generated. The FPKM of each gene was calculated, and the read counts of each gene were obtained using HTSeq‐count. PCA was performed using R (v 3.2.0) to evaluate duplicates. Genes with a Q value < 0.05, fold change > 2 or < 0.5 were considered as significantly differentially expressed, and differential expression analysis was performed using DESeq.

### scRNA‐seq Analysis

Publicly available scRNA‐seq data was retrieved through the GEO database under accession code “GSE189381” for the analysis of mouse pulp P7.5.^[^ ^20^ ^]^

### Neutralization Experiment

Mouse pups of *Ctsk‐Cre;tdTomato* were injected intraperitoneally with 5 µmol Anti‐Sonic Hedgehog antibody (ab19897, Abcam) each day for five days from P3.5 to P7.5 and harvested at P8.5. The neutralization efficiency was confirmed via immunostaining before subsequent analyses.

### Statistics Analysis

All measurements were performed by a blinded examiner in at least three independent trials, and the average was calculated and documented. All data were analyzed as mean ± SEM. For analysis of the statistical significance of differences between two groups, two‐tailed Student's t‐tests were generally performed. For analysis of the statistical significance of differences among more than two groups, it performed repeated measures one‐way ANOVAs and Tukey's multiple comparisons tests. A *P*‐value < 0.05 was considered statistically significant. All statistical tests were performed using GraphPad with Prism9, following its statistics guide.

## Conflict of Interest

The authors declare no conflict of interest

## Author Contributions

R.X., Q.Y., and C.Z. performed conceptualization. R.X., X.Z., Y.W., D.Z., S.J., L.L., J.W., and W.L. performed methodology. R.X., X.Z., D.Z., S.J., L.L., W.L., and X.L. performed investigation. R.X., X.Z., D.Z., S.J., L.L., and J.J. performed visualization. Q.Y., C.Z., and J.J. performed supervision. R.X., X.Z., Y.W., D.Z., S.J., L.L., W.L., and J.J. wrote the original draft. R.X., X.Z., Q.Y., and C.Z. wrote, review, and edited the final manuscript. All authors reviewed and approved the manuscript.

## Supporting information



Supporting Information

## Data Availability

The data that support the findings of this study are available in the supplementary material of this article.
