# Supplementary material for: Cathepsin K‐Positive Cell Lineage Promotes In Situ Dentin Formation Controlled by Nociceptive Sonic Hedgehog
Source: Adv Sci (Weinh). 2024 Oct 30;11(47):2310048. doi: 10.1002/advs.202310048 (PMC11653649; doi:10.1002/advs.202310048)
Supplement: Supplementary file 1 — Supporting Information [file ADVS-11-2310048-s001.pdf]

ADVANCED SCIENCE
Open Access

Supporting Information

for *Adv. Sci.*, DOI 10.1002/advs.202310048

Cathepsin K-Positive Cell Lineage Promotes In Situ Dentin Formation Controlled by Nociceptive Sonic Hedgehog

Ruoshi Xu, Xiaohan Zhang, Weimin Lin, Yushun Wang, Danting Zhang, Shuang Jiang, Linfeng Liu, Jiaying Wang, Xutao Luo, Xiao Zhang, Junjun Jing, Quan Yuan and Chenchen Zhou**

Supplementary Text and Figures:

Developmental and aging cathepsin k-positive lineage tilts towards in situ dentin regeneration controlled by nociceptive sonic hedgehog

The following includes:

1. Supplementary Fig. 1 to Supplementary Fig. 7
2. Supplementary Table. 1

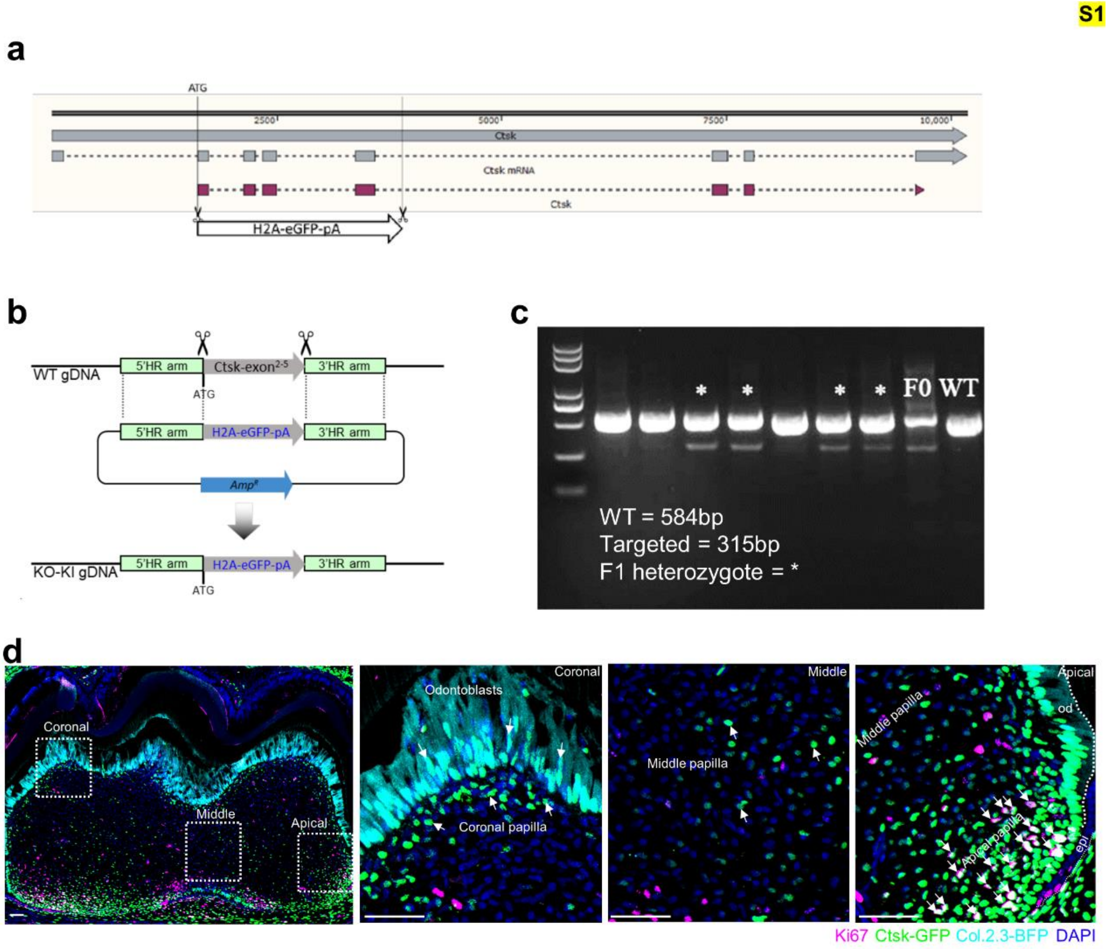

Supplementary Fig. 1 Generation of *Ctsk-GFP* mice.

a-b Schematic representation of the *Ctsk-GFP* mice strategy. **c** PCR genotyping and Southern blot examination of the *Ctsk-GFP* mice. The specific primers and PCR procedure have been provided in Supplementary Table 1. Protocols for genotyping of

Ctsk-GFP. **d** Ki67 immunostaining on Cryostat section of *Ctsk-GFP;Col2.3-BFP* mice molar at P7.5 to outline boundary of odontoblast (recolored by cyan) from coronal, middle and apical papilla Ctsk-GFP^{+} cells. Scale bar:50μm.

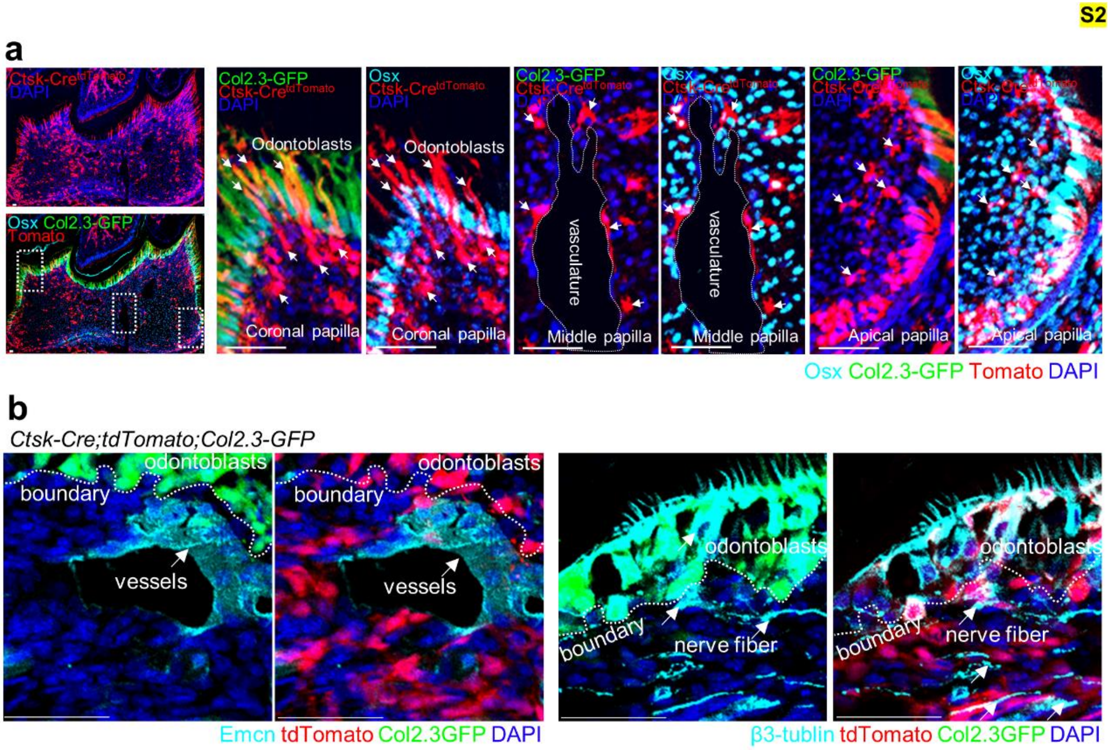

Supplementary Fig.2 Tracing of *Ctsk-Cre;tdTomato;Col2.3-GFP* mice

a Cryostat section of *Ctsk-Cre;tdTomato;Col2.3-GFP* mice molar at P7.5 with immunostaining using Osx antibody. tdTomato labeled Ctsk^{+} lineage in odontoblasts (Col2.3-GFP^{+} labeled), middle and apical papilla (arrow). **a** Cryostat section of *Ctsk-Cre;tdTomato;Col2.3-GFP* mice molar at 1mon with immunostaining using Emcn and β3-tublin antibody. Boundary of odontoblast, vessels and nerve fibers were indicated. Scale bar:50μm.

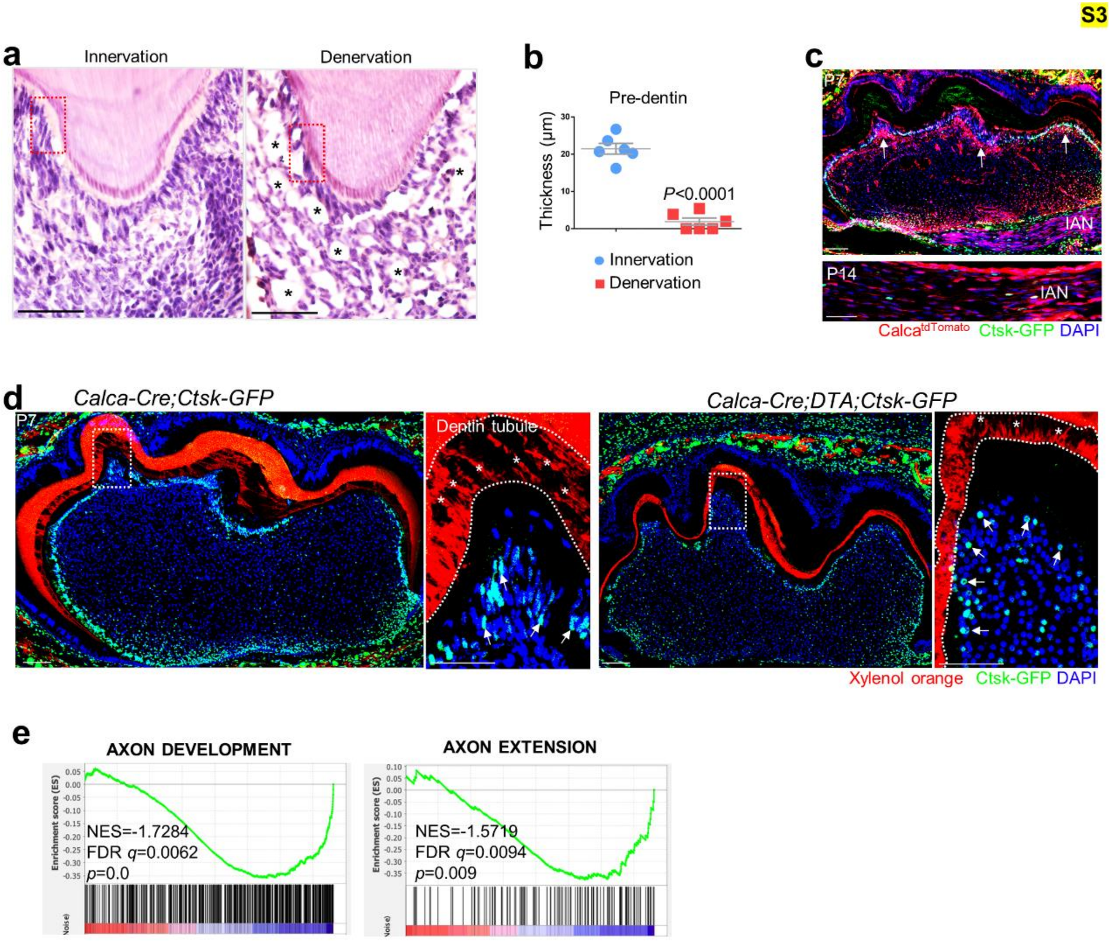

Supplementary Fig.3 Loss of nerve reduced odontogenesis and downregulated Hedgehog.

a H&E staining indicated impaired pre-dentin formation. Framed areas are shown in Fig.**3c**. Scale bar, 20μm. **b** Quantification of predentin thickness (n=6 mice). **c** Cryostat section of *Calca-Cre;tdTomato;Ctsk-GFP* mice molar at P7 and IAN at P14. Tomato-labeled nociceptive associated cells and IAN nerve were indicated by arrows. **d** Xylenol orange staining on cryostat section of *Calca-Cre;DTA;Ctsk-GFP* mice molar at P7. Dentin (dotted line) with tubules(asterisk) and *Ctsk-GFP* signals were indicated. **e** GSEA analysis indicated downregulation of axon development. Scale bar:50um. Data are shown as the mean ± S.E.M, unpaired two-tailed Student's t-test.

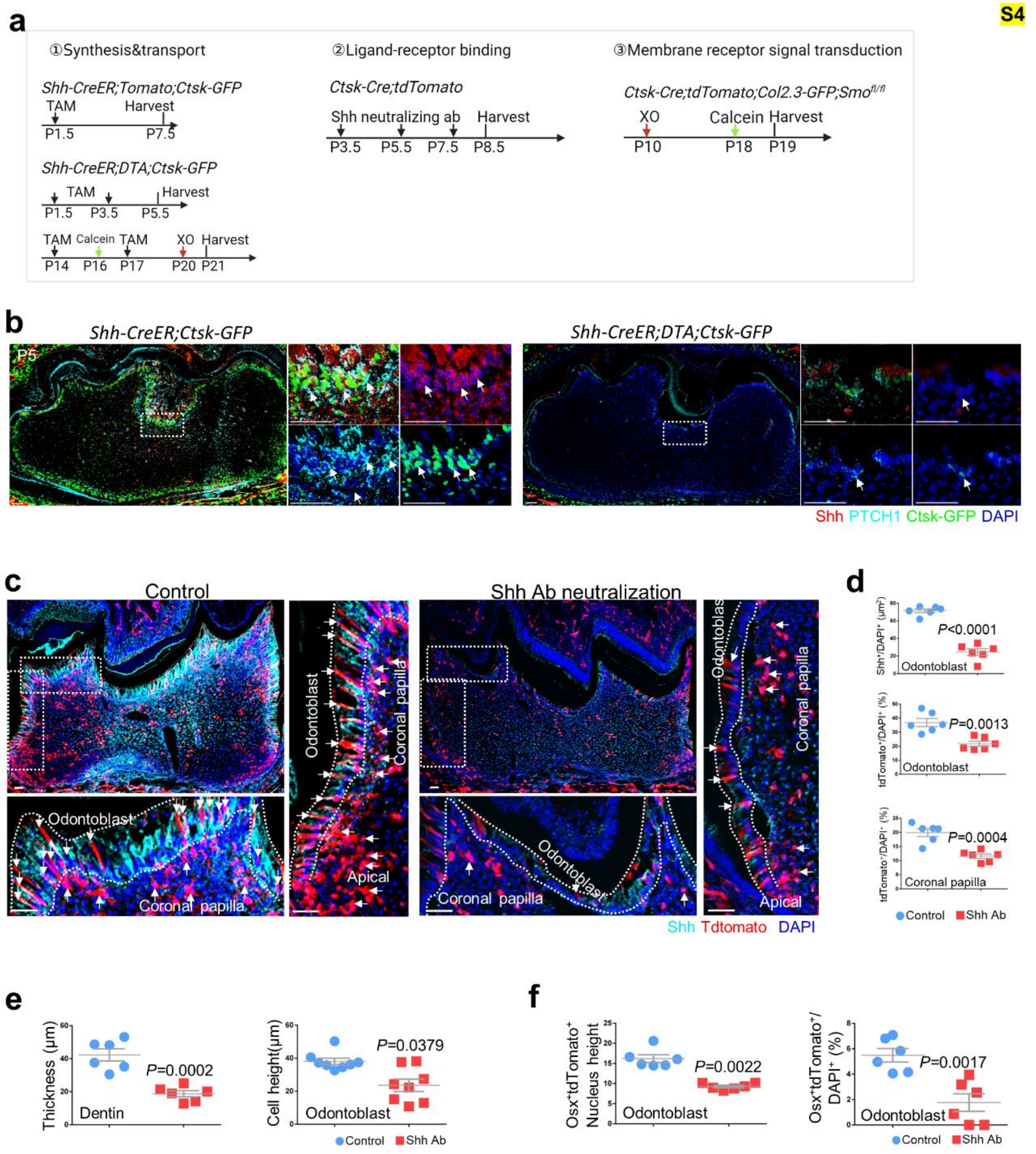

Supplementary Fig.4 Efficiency and quantification for loss of Shh signaling.

a Schematics of blocking Shh signaling. **b** Immunostaining of indicated antibody on *Shh-CreER;DTA;Ctsk-GFP* mice at P5.5. **c** Efficiency of Shh antibody neutralization and reduced Ctsk^{+} lineage on *CtskCre;tdTomato* mice. **d** Quantification of the percentage of *tdTomato*$^{+}$ cells versus DAPI^{+} cells in **c**. (n=6 fields from three mice). **e** Quantification of dentin thickness and odontoblast height in Masson staining of Fig.**4e** (n=6 fields from three mice). **f** Quantification of nucleus height of Osx/*tdTomato* double positive cells in immunostaining of Fig.**4e** (n=6 fields from three mice). Scale bar:50um. Data are shown as the mean ± S.E.M, unpaired two-tailed Student's t-test.

S5

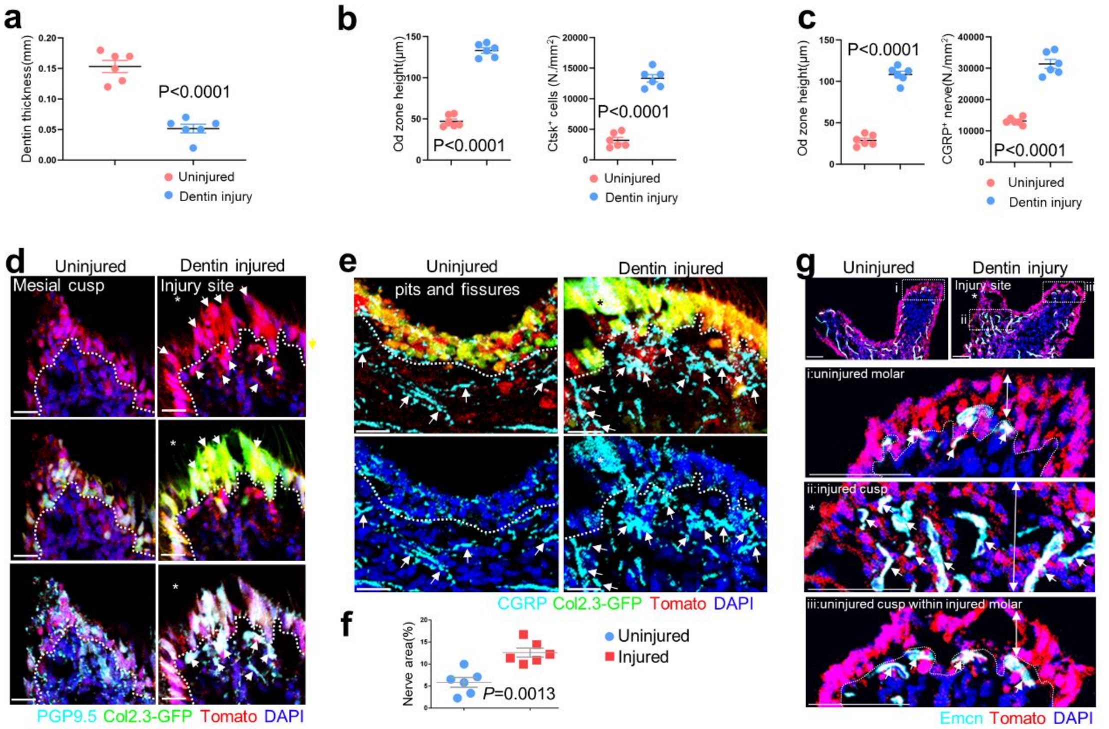

Supplementary Fig. 5 Dentin injury trigger nerve ingrowth and odontoblasts elongation.

a Quantification of dentin thickness of Fig.**5c**. **b** Quantification of odontoblast zone length and Calca^{+} nerve of Fig.**5e**. **c** Quantification of odontoblast zone length and Ctsk^{+} cells of Fig.**5f**. **d** Immunostaining using PGP9.5 on cryostat section of *Ctsk-CreER;tdTomato;Col2.3-GFP* mice molars one week after the mesial cusp injury (asterisk). Elongated odontoblasts (arrows above dotted line) and increased transiting nerve(arrows beneath dotted line) were indicated. **e** Immunostaining using CGRP antibody on cryostat section of *Ctsk-CreER;tdTomato;Col2.3-GFP* mice molars one week after the injury. Nociceptive nerve fibers (arrows beneath dotted line) was increased beneath elongated odontoblasts (dotted line). **f** Increased nerve transiting was quantified (n=6 mice). **g** Immunostaining using Emcn antibody on cryostat section of *Ctsk-CreER;tdTomato* mice molars one week after the mesial cusp injury. vessels increased(arrows) and odontoblasts elongation (double arrows) were indicated. Scale bar:50um. Data are shown as the mean ± S.E.M, unpaired two-tailed Student's t-test.

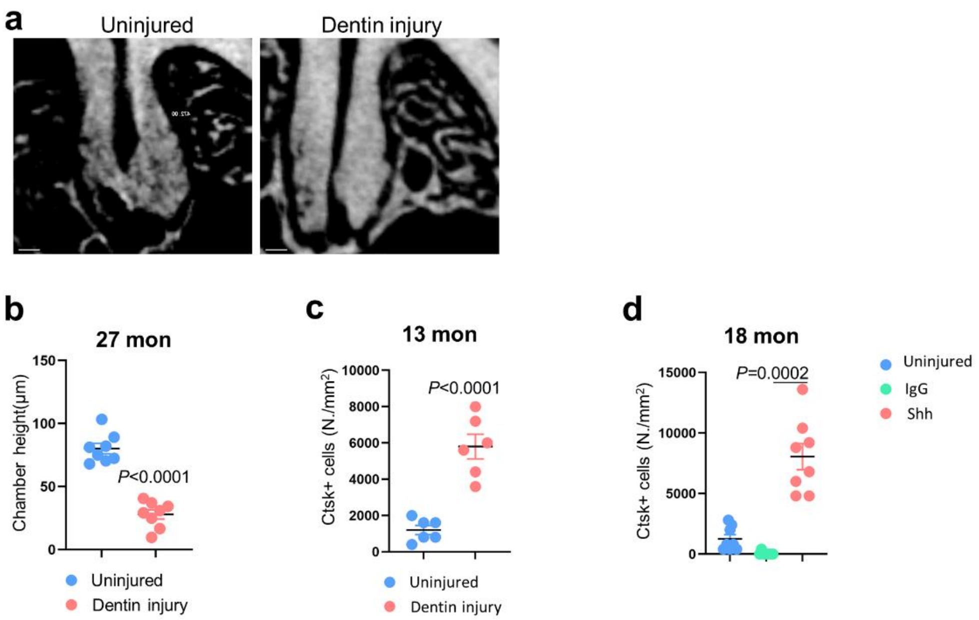

Supplementary Fig.6 Quantification for aged mouse model.

a 2D sagittal view of root of uninjured and injury mouse molars at the age of 27 months, supplementary to Fig.**8c**. **b** Quantification of chamber height changes due to reactionary dentin formation of uninjured and injury mouse molars at the age of 27 months, supplementary to Fig.**8c**. (n=8 mice). **c** Quantification of Ctsk-GFP^{+} cells of uninjured and injury mouse molars at the age of 13 months, supplementary to Fig.**8d**. (n=6 fields from four mice). **d** Quantification of Ctsk-GFP^{+} cells surrounding GSIB^{+} vasculature of uninjured molar group, injured molar with IgG+BP group, injured molar with SHH+BP group on Ctsk-GFP mouse at the age of 18 months, supplementary to Fig.**8e**. (n=8 fields from four mice). Data are shown as the mean ± S.E.M, unpaired two-tailed Student's t-test. Scale bar, 100um.

S7

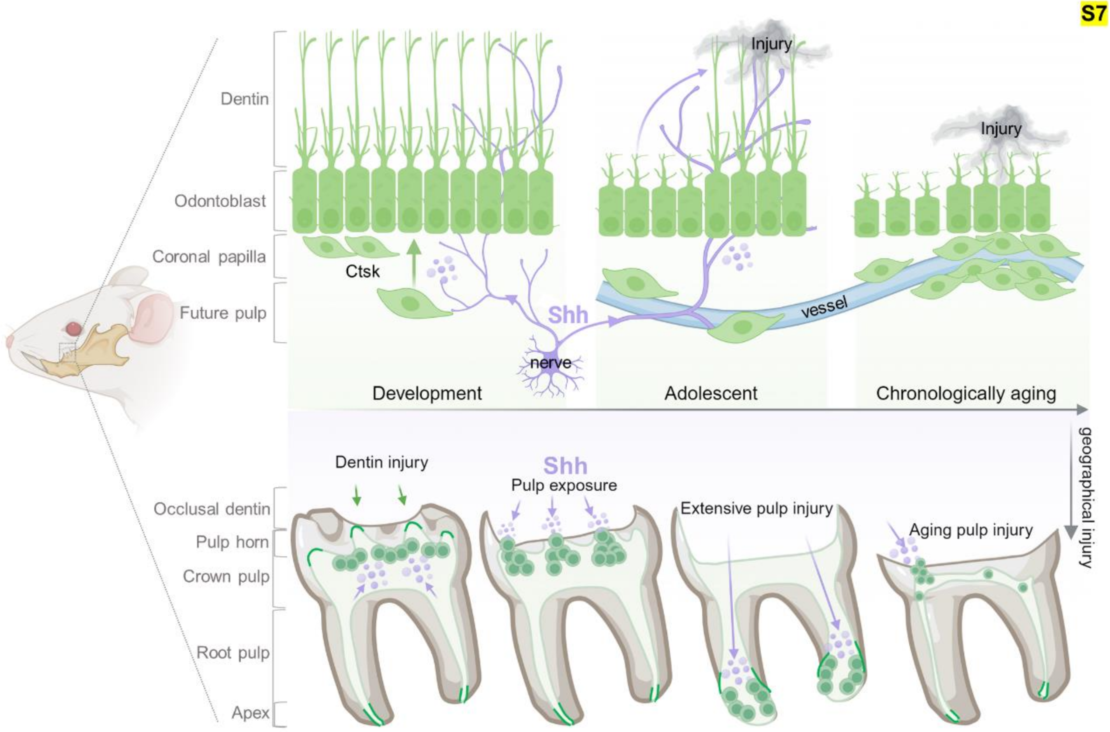

Supplementary Fig.7 Schematics of the Ctsk^{+} lineage and its mechanism for molar odontogenesis.

Chronologically, Ctsk^{+} lineage is required for odontogenesis and activated by the nerve-derived Shh and regulated by Hedgehog signaling pathway from neonatal development to aging. Dentin injury triggers Ctsk^{+} lineage for reactionary odontogenesis regulated by endogenous nerve-derived Shh. Exogenous Shh to progressive geological pulp injury site could promote reparative odontogenesis and apex development. In particular, the aging pulp still hold the capacity to promote reparative odontogenesis, which is supported by aging Ctsk^{+} lineage exogenously controlled by local Shh administration.

Supplementary Table1 :
Protocols for genotyping of *Ctsk-GFP*

➢ **Primers for genotyping of *Ctsk-GFP***

Primer Type	Sequence 5'→3'
Wild type Forward	AGTTTTCTCAGGCTGGCAGA
Mutant Forward	GGGAGGATTGGGAAGACAAT
Common Reverse	GGATAGCCATGTCTGGAACC

➢ **PCR program for genotyping of *Ctsk-GFP***

STEP	TEMP℃	TIME	NOTE
1	95.0	3min	
2	95.0	30sec	
3	59.0	30sec	
4	72.0	1min	
5	--	--	repeat steps 2-4 for 30 cycles
6	72.0	2min	
7	10.0	--	hold
